# Prevalence and Patterns of Refractive Error Among School-Age Children in Bisha, Saudi Arabia

**DOI:** 10.7759/cureus.50530

**Published:** 2023-12-14

**Authors:** Abdulmajeed Alkhathami, Saad Ali M Alqarni, Amal T Aljuaid, Yazeed F Alshahrani, Jehad Alabdulminaim, Yousef Al-Otaibi, Mutasim E Ibrahim

**Affiliations:** 1 College of Medicine, University of Bisha, Bisha, SAU; 2 College of Medicine, Majmaah University, Al Majma'ah, SAU; 3 Department of Ophthalmology, College of Medicine, University of Bisha, Bisha, SAU; 4 Department of Medical Education, College of Medicine, University of Bisha, Bisha, SAU; 5 Department of Microbiology, College of Medicine, University of Bisha, Bisha, SAU

**Keywords:** saudi arabia, bisha, prevalence, school-age children, refractive error

## Abstract

Background: A widespread and serious eye condition is a refractive error (RE). Globally, uncorrected refractive defects affect numerous individuals, with some who are blind. Numerous studies in Saudi Arabia have been conducted to assess reflective error. However, there is a lack of knowledge regarding RE among school-age children in Bisha province, southwest Saudi Arabia. This study aimed to determine the prevalence and types of RE among school-age children in Bisha, Saudi Arabia.

Methods: A cross-sectional study involved 360 schoolchildren from primary schools was carried out between December 2022 and November 2023 in Bisha. A validated questionnaire form was used to collect sociodemographic information and clinical data (history of the ocular problem, visual acuity test findings, and the refractor machine's result).

Result: A total of 360 schoolchildren aged from seven to 14 years, with a mean of 10.1 years (standard deviation (SD)=2.05). The prevalence of hyperopia was 21% in the right eye and 23% in the left eye. In addition, the prevalence of myopia was 20% in the right eye and 22.5% in the left eye. A significant association between visual acuity and myopia (p=0.001). By contrast, there was no significant correlation between hyperopia and visual acuity (p=0.412).

Conclusion: The current study summarized the prevalence of REs among school-age children in Bisha, Saudi Arabia. The study population included nearly half of those with at least some degree of RE. These results highlight the need for prompt and careful screening programs to detect and treat refractive disorders across this age range.

## Introduction

A preventable blindness condition is a refractive error (RE). According to the World Health Organization (WHO), in 2006, approximately 153 million people over the age of five worldwide suffer from uncorrected refractive abnormalities; eight million of these people are blind [1]. Furthermore, 12.8 million persons worldwide, or 0.96% of the population between the ages of five and 15, suffer from vision impairment as a result of uncorrected or badly corrected refractive abnormalities; Southeast Asia and China show the greatest occurrence rates in metropolitan and highly developed areas [2]. Many research has been carried out in Saudi Arabia to evaluate the RE and other eye problems of the country's population.

According to a study done in Jazan, south Saudi Arabia, astigmatism (31%), hyperopia (32.2%), and myopia (17.2%) were the most common REs. Mixed astigmatism (3.5%) and hyperopic astigmatism (16.1%) came next [3-5]. In the same region, another study found that the prevalence of hyperopia was 3.6% in healthy people and 27.6% in those with RE. Amblyopic eyes made up 30% of hyperopic eyes. Seventy percent of students had RE, while 9.3% of students had myopia. Amblyopic eyes made up just 9% of myopic eyes [4]. According to a study, myopia accounted for 24.4% of cases of RE in Arar, northern Saudi Arabia, with hyperopia accounting for 11.9% and astigmatism for 9.5% of cases. However, in a different study conducted in the nation, it was discovered that gender and age had a substantial impact on the prevalence of the various patterns of RE [6]. According to a cross-sectional study done in Al Hassa, the population under study had a prevalence of myopia that explained 9.0% of the hypermetropia that was found in 27 students, 1.4% of myopic 34, and hyperopic astigmatism 33 [7].

Previous research found a substantial correlation between the development of myopia and older age, as well as higher academic standing in the previous semester [3]. However, school-age youngsters in Bisha province, southwest Saudi Arabia, do not know anything about RE. Early identification of these health issues may help limit the subsequent impact they have on school-age children.

This study aimed to determine the prevalence and types of RE among school-age children in Bisha, Saudi Arabia. In addition, the study sought to investigate the common risk factors associated risk associated with RE.

## Materials and methods

A descriptive questionnaire-based cross-sectional study was carried out during the period from December 2022 to November 2023 in Bisha province, southwest Saudi Arabia. All the governmental schools in the province were involved in a computer list that the Ministry of Education accessed; then, we used a computer to choose four schools randomly. Schoolchildren from both genders were selected from four primary public schools to participate in the study. The selected schools (two males and two females) are considered the main governmental public schools in Bisha. These are Angal Bisha Primary School, King Abdullah School, Bright International School Bisha, and 12th Elementary Female Primary School. Schoolchildren who were involved in our study were selected through a simple random sampling technique. 

Sample size

Using the Richard Geiger equation (n0=Z2pq/d2), with a margin of error determined as 5%, a confidence level of 95%, and a 50% response distribution, to get the final total sample size, which equals to 288 children. We added 25% to avoid bias in the number of participants, so 360 children were involved in our study.

Inclusion and exclusion criteria

All schoolchildren aged from seven to 14 years old were included in the study. Children who were physically disabled, previously diagnosed with a vision problem, and psychologically ill were excluded. 

Data collection and procedure

A validated questionnaire form was used to collect information about sociodemographic characteristics and clinical data on the history of the ocular problem, findings of the visual acuity test, cover-uncover test, and the result of the refractor machine.

Procedure

The results of the refractor machine were broken down into many stages: A certified optometrist conducted a 10-minute vision test as a part of the medical evaluation. Teens with an abnormal ocular movement, an eye problem (strabismus, nystagmus, or ptosis), or a visual acuity of 6/9 (20/28) or worse in one or both eyes were referred for a fuller 45-minute ophthalmic examination within a month. This examination included the following tests: an auto chart projector (ACP-8 Series, Topcon Corporation, Tokyo, Japan) and the Snellen "Tumbling E" eye chart were utilized to measure each student's uncorrected visual acuity fully. Six meters will separate them from the brightly lit Snellen chart. Every eye was tested independently for visual acuity. The pupil was able to read more than half of the letters on the line with the lowest font; therefore, it was noted.

Cover-Uncover Test

A cover-uncover test was used to measure eye alignment at near (40 cm) and far (3 m) distances. During the exam, the screener covered the student's left eye with a paddle and instructed the student to focus on a certain, standardized fixation target. We held the paddle in front of the eye for around three seconds. In order to ascertain whether refixation took place, the screener examined the unhindered right eye. At least three iterations of the cover-uncover test were conducted. A tabletop video/photo refractor, the Power Refractor II (version 3.11.01.24.00), evaluated eye alignment and the RE in eight meridians binocularly.

Statistical methods

Data were collected, arranged, and entered in an Excel sheet (Microsoft, USA) and then transferred to IBM SPSS Statistics for Windows, version 25 (released 2017; IBM Corp., Armonk, New York, United States). Descriptive statistics were carried out and presented as mean, standard deviation, proportions, and frequency tables. Every two variables were compared using the chi-square test. P-values were considered statistically significant if they had a value less than 0.05.

Ethical approval

Before completing the surveys, each parent was shown the purpose and significance of the study and asked for a written agreement. The participants' confidentiality was kept as the questionnaires contained no personal data referring to or implying the participants’ identity. All of the participants included in the study were anonymous.

The University of Bisha College of Medicine's (UBCOM) ethical approval granted clearance (ref. no. UB-RELOC H -06-BH-087/ (0905.23)).

## Results

A total of 360 schoolchildren from primary schools were enrolled in the study. The age of the participants ranged from seven to 14 years, with a mean of 10.1 years (standard deviation (SD)=2.05). Most of the participants were boys (59.4%, n=214). About half of the participants are in age equal to or more than 10 years. Most participants were from urban areas (86.1%, n=310). 

Of the 360 pupils, the majority were from year five (22.2%, n=80) and year three (20.6%, m=74), followed by year six (16.7%, n=60) and year two (15.6%, n=56) (Table 1). 

**Table 1:** General characteristics of the participants

Variable	n (%)
Gender	
Male	214 (59.4)
Female	146 (40.6)
Age group (in years)	
Less than 10	173 (48.1)
Equal or more than 10	187 (51.9)
Residence	
Urban	310 (86.1)
Rural	50 (13.9)
Educational level	
First year	44 (12.2)
Second year	56 (15.6)
Third year	74 (20.6)
Fourth year	46 (12.8)
Fifth year	80 (22.2)
Sixth year	60 (16.7)
Spectacles used by students	
Yes	38 (10.6)
No	322 (89.4)
Spectacles used by siblings	
Yes	120 (33.3)
No	240 (66.7)

**Figure 1:**
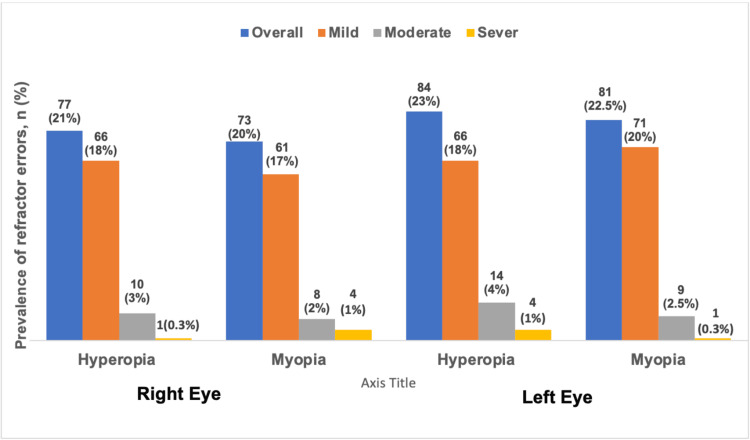
Prevalence and classification (high, moderate, and mild) of refractive errors in both the left and right eyes of the participants.

Among the 360 pupils, the prevalence of hyperopia was 21% in the right eye and 23% in the left eye. In addition, the prevalence of myopia was 20% in the right eye and 22.5% in the left eye (Figure 1).

**Figure 2:**
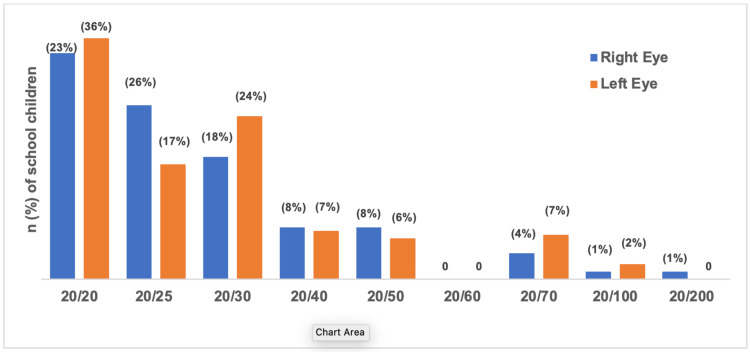
Findings of visual equity measurements of the right and left eyes of schoolchildren

Figure 2 illustrates the percentage of visual acuity of the participants. In our study, we define myopia and hyperopia in dioptres (D) as follows: mild myopia (-0.5 to less than -3 D), moderate myopia (-3 to less than -6 D), and severe myopia (greater than -6 D); mild hyperopia (+0.5 to less than +3D), moderate hyperopia (+3 to less than + 6D), and severe hyperopia (greater than +6 D). Most of the pupils had a normal visual acuity in the right eye of 20/20 (n=122, 33.9%); the lowest result was 20/100 and 20/200 (n=4, 1.1%). On the other hand, the visual acuity of the left eye was normal at 20/20 in 130 participants (36.1%); the least visual acuity was 20/100 in eight participants (2.2%). 

Table 2 presents the association between the visual acuity and baseline characteristics of the students. There is a significant association between visual acuity of the right eye and age group (p=0.036) and residency (p=0.028) of students. Pupils 10 years or above and residing in local areas were more likely to have squints in the left eye. In addition, those who had previous health problems were more likely to have abnormal acuity in the left eye (p=0.028).

**Table 2:** Association between the visual acuity and baseline characteristics of the participants

Variable	Normal visual acuity of the right eye			Normal visual acuity of the left eye		
	Yes	No	P value	Yes	No	P value
Age group			0.036			0.445
<10	76 (44)	97 (56)		64 (37)	109 (63)	
≥10	62 (33)	125 (67)		62 (33)	125 (67)	
Gender			0.381			0.804
Male	86 (40)	128 (60)		76 (35.5)	138 (64.5)	
Female	52 (36)	94 (64)		50 (34)	96 (66)	
Residence			0.032			0.873
Urban	112 (36)	198 (64)		108 (35)	202 (65)	
Rural	26 (52)	24 (48)		18 (36)	32 (64)	
School level			0.281			0.563
1^st^ year	16 (36)	32 (64)		14 (32)	30 (68)	
2^nd^ year	24 (43)	38 (57)		18 (32)	38 (68)	
3^ed^ year	36 (49)	38 (51)		32 (43)	42 (57)	
4^th^ year	16 (35)	30 (65)		14 (30)	32 (70)	
5^th^ year	28 (35)	52 (65)		30 (37.5)	50 (62.5)	
6^th^ year	18 (30)	42 (70)		18 (30)	42 (70)	
Previous ocular problem						0.028
Yes (No)	20 (29)	48 (71)	0.093	16 (23)	52 (77)	
	118 (40)	174 (60)		110 (38)	182 (62)	

Table 3 presents the association between the type of RE and the general characteristics of the students. Hyperopia and myopia did not show insignificant associations related to the gender, age group, and educational levels of the schoolchildren. However, there was a significant association between myopia and residence (p=0.026), where the disorder was higher among students from urban areas. In addition, students with previous ocular problem were more likely to have hyperopia than those without ocular problems (p=0.004).

**Table 3:** Association between the type of refractive error and baseline characteristics of the students

Variable	Hyperopia				Myopia			
	Mild	Moderate	Sever	P value	Mild	Moderate	High	P value
Age group				0.15				0.27
<10	53 (31)	12 (7)	4 (2)		33 (19)	5 (3)	4 (2)	
≥10	50 (27)	3 (2)	1 (0.5)		45 (24)	7 (4)	1 (0.5)	
Gender				0.63				0.37
Male	60 (28)	8 (4)	4 (2)		48 (22)	5 (2)	4 (2)	
Female	43 (29)	7 (5)	1 (1)		30 (21)	7 (5)	1 (1)	
Residence				0.97				0.026
Urban	93 (30)	9 (3)	5 (2)		71 (23)	12 (4)	5 (2)	
Rural	10 (20)	6 (12)	0 (0)		7 (14)	0 (0)	0 (0)	
School level				0.54				0.277
1^st^ year	12 (27)	6 (14)	0 (0)		13 (29.5)	0 (0)	0 (0)	
2^nd^ year	15 (27)	4 (7)	2 (4)		12 (21)	2 (4)	2 (4)	
3^ed^ year	25 (34)	2 (3)	2 (3)		8 (11)	3 (4)	2 (3)	
4^th^ year	9 (20)	2 (4)	0 (0)		17 (37)	2 (4)	0 (0)	
5^th^ year	24 (30)	1 (1.25)	0 (0)		14 (17.5)	5 (6)	1 (1.25)	
6^th^ year	18 (30)	0 (0)	1 (2)		14 (23)	0 (0)	0 (0)	
Previous ocular problem								
Yes (No)	25 (37)	6 (9)	2 (3)	0.004	14 (21)	7 (10)	1 (1.5)	0.922
	78 (27)	9 (3)	3 (1)		64 (22)	5 (2)	4 (1)	

Table 4 summarizes the association between the type of REs and visual acuity. There was a significant association between visual acuity and myopia (p=0.001). Meanwhile, there was no significant correlation between hyperopia and visual acuity (p=0.412).

**Table 4:** Association between visual acuity and type of refractive error

Visual acuity	Hyperopia			P value
	Mild	Moderate	Sever	
Right eye				
Normal	39 (38)	9 (60)	3 (60)	0.412
Abnormal	64 (62)	6 (40)	2 (40)	
Left eye				
Normal (abnormal)	39 (38)	3 (20)	5 (100)	
	64 (62)	12 (70)	0	
Visual acuity	Myopia			P value
	Mild	Moderate	Sever	
Right eye				
Normal	16 (21)	1 (8)	0	
Abnormal	62 (79)	11 (92)	5 (100)	0.001
Left eye				
Normal (Abnormal)	11 (14)	1 (8)	0	
	67 (86)	11 (92)	5 (100)	

## Discussion

In this study, the prevalence of REs among schoolchildren in Bisha, Saudi Arabia, between the ages of seven and 14 years, was compared to reports from other parts of the world. Understanding that RE is a complicated and multidimensional condition with a wide range of genetic, demographic (age, race, ethnicity, and geography), ocular, and extrinsic factors (pressure to pursue higher education, changes in lifestyle, and prolonged indoor and near activities) variations in its prevalence. Numerous studies have evaluated the frequency of different types of refractive defects in students [4-8]. To enable meaningful comparison, we restricted the comparison to a study that was published in 2000 and after [9-14]. The overall prevalence in our study was very high; with 55.8% of all participants, it was significantly associated with age, residence, history of previous ocular problems, and visual acuity test. A high prevalence of REs in schoolchildren can impose a significant economic burden on families and healthcare systems. The cost of eye examinations, eyeglasses, contact lenses, and other vision correction interventions can be substantial, particularly for families with limited financial resources. However, a history of squint was not common among the participants. Few of the students were using spectacles. 

Uncorrected RE can damage a person's vision in both children and adults, with both short- and long-term consequences, such as missed chances for school and employment; slower economic growth for individuals, families, and countries; and a lower standard of living [8]. REs go uncorrected for a variety of reasons, including lack of awareness and recognition of the issue at the individual, family, community, and public health levels; lack of accessibility to and/or affordability of refractive testing services; inadequate availability of reasonably priced corrective lenses; and cultural barriers to compliance [9]. Although refractive defects might be easily repaired, the estimated amount of visual impairment brought on by uncorrected REs is of public health concern [6].

RE is more common in some regions than others, ranging from 10% in Australia [3] to 50.3% in India [9]. In our study, there was a high prevalence of REs, which is similar to the study done in Taif region of Saudi Arabia, with a 50.9% prevalence rate. This is in contrast to what was reported in King Abdul-Aziz Medical City, Riyadh (9.8%) [5,14], Al-Hassa, eastern Saudi Arabia (13.7%) [7], and Qassim Province, Saudi Arabia (16.3%) [11,14]. Even among studies carried out in the same geographic area, there is a significant variation in the prevalence of RE overall. This wide variation may be due to variations in the operational definition, cut-off values used to identify various types of REs, and measurement techniques. 

It has been discovered that the prevalence of hyperopia varies significantly among different people [1,8]. The current study stated that the prevalence of hyperopia was higher (32.2%) than that previously reported from Saudi Arabia (0.7-17.63%) [11,14]. In addition, our study showed an increased tendency for hyperopia prevalence among those aged less than 10 years (62%) compared to those aged more than 10 years (54%), which is inconsistent with findings in Riyadh's study, where only 4.7% of them had hyperopia [15].

Similar surveys conducted in schools across the globe reveal that myopia prevalence varies from 0.8% to 65.7%. In the current study, myopia was detected in 26.9% of the individuals, which is similar to earlier findings from Al-Hassa, Saudi Arabia (23.4%) [7], Taif, Saudi Arabia (33.2%) [2], China (36.9%) [1], and Iran (29.3%) [10,16]. However, it is higher than those previously reported in Al-Qassim (5.8%) and Riyadh (5.7%). Given the global trend of myopia increasing, prevention programs, including limiting near activities and encouraging children to participate in more outdoor activities, may be necessary.

Study limitations

In order to highlight the necessity of establishing a school-based child eye care system in Saudi Arabia, the primary goals of this study were to estimate the prevalence of RE and compare the findings with those of similar studies conducted in Saudi Arabia and other nations. This research, however, necessitates additional studies of a similar nature that concentrate on the causes, risk factors, and associations between various REs, as these aspects will aid in the rationale and explanation of the findings and development of necessary preventive measures that will give future research greater weight.

In addition to the high rate of student absenteeism and gender-related concerns, this study was conducted around the conclusion of the academic year, which is a very limited period to perform such a significant study with a large sample. Children with attention-deficit hyperactivity disorder (ADHD), autism, or other conditions that could interfere with their ability to cooperate and comprehend the tests being administered were not allowed. The lack of transportation in more rural locations also prevented schools from being included.

## Conclusions

The prevalence of REs among school-age children in Bisha, Saudi Arabia, was presented in the current study. The study results showed that half of the sample population in this area had at least some REs. According to the study's findings, about half of the participants in this research had some degree of REs. These results highlight the need for prompt and careful screening programs to detect and treat refractive problems in this age range.
